# The effect of terrain on the fine‐scale genetic diversity of sub‐Antarctic Collembola: A landscape genetics approach

**DOI:** 10.1002/ece3.11519

**Published:** 2024-06-18

**Authors:** Daniela Marques Monsanto, David William Hedding, Sandra Durand, Shilpa Pradeep Parbhu, Matthew Grant Adair, Arsalan Emami‐Khoyi, Peter Rodja Teske, Bettine Jansen van Vuuren

**Affiliations:** ^1^ Department of Zoology, Centre for Ecological Genomics and Wildlife Conservation University of Johannesburg Auckland Park South Africa; ^2^ Department of Geography University of South Africa Pretoria South Africa; ^3^ Institute of Wildlife Management and Nature Conservation Hungarian University of Agriculture and Life Sciences Gödöllő Hungary

**Keywords:** landscape resistance, microsatellites, Prince Edward Islands, springtail, sub‐Antarctic

## Abstract

Biodiversity patterns are shaped by the interplay between geodiversity and organismal characteristics. Superimposing genetic structure onto landscape heterogeneity (i.e., landscape genetics) can help to disentangle their interactions and better understand population dynamics. Previous studies on the sub‐Antarctic Prince Edward Islands (located midway between Antarctica and Africa) have highlighted the importance of landscape and climatic barriers in shaping spatial genetic patterns and have drawn attention to the value of these islands as natural laboratories for studying fundamental concepts in biology. Here, we assessed the fine‐scale spatial genetic structure of the springtail, *Cryptopygus antarcticus travei*, which is endemic to Marion Island, in tandem with high‐resolution geological data. Using a species‐specific suite of microsatellite markers, a fine‐scale sampling design incorporating landscape complexity and generalised linear models (GLMs), we examined genetic patterns overlaid onto high‐resolution digital surface models and surface geology data across two 1‐km sampling transects. The GLMs revealed that genetic patterns across the landscape closely track landscape resistance data in concert with landscape discontinuities and barriers to gene flow identified at a scale of a few metres. These results show that the island's geodiversity plays an important role in shaping biodiversity patterns and intraspecific genetic diversity. This study illustrates that fine‐scale genetic patterns in soil arthropods are markedly more structured than anticipated, given that previous studies have reported high levels of genetic diversity and evidence of genetic structing linked to landscape changes for springtail species and considering the homogeneity of the vegetation complexes characteristic of the island at the scale of tens to hundreds of metres. By incorporating fine‐scale and high‐resolution landscape features into our study, we were able to explain much of the observed spatial genetic patterns. Our study highlights geodiversity as a driver of spatial complexity. More widely, it holds important implications for the conservation and management of the sub‐Antarctic islands.

## Graphical abstract

The interplay between geodiversity and organismal characteristics shapes biodiversity patterns and by superimposing genetic structure on landscape heterogeneity, one can disentangle these interactions to better understand population structure and dynamics. The sub‐Antarctic Islands offer unique opportunities to study fundamental concepts in biology given their heterogeneous landscapes shaped by repeated large‐scale volcanic and glacial events. The present study focuses on the springtail *Cryptopygus antarcticus travei*, a soil arthropod endemic to Marion Island and uses a species‐specific suite of microsatellite markers and a fine‐scale sampling design incorporating landscape complexity. This study illustrates that the fine‐scale genetic patterns in soil arthropods are markedly more structured than anticipated, given the homogeneity of the vegetation complexes at the scale of tens to hundreds of metres. This work adds valuable new information to better understand the effects of local‐scale landscape heterogeneity on the population genetic structure of the island's unique biota.

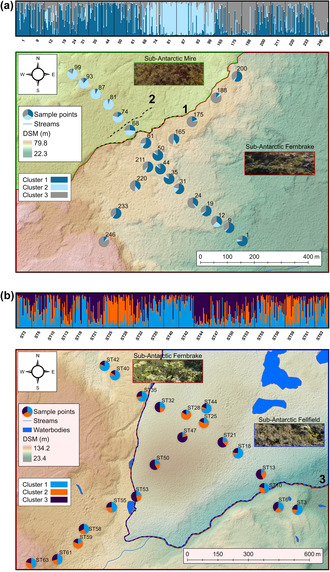

## 1 INTRODUCTION

Biodiversity is structured by the relationship between landscape heterogeneity (e.g., inter‐habitat distance, habitat composition and landscape barriers) and organismal characteristics (e.g., reproductive strategies and dispersal capabilities) (Henriques‐Silva et al., 2015; Nathan, 2008). Manel et al. (2003) defined landscape genetics as an amalgam of landscape ecology and population genetics that uncovers the relationship between landscape heterogeneity and microevolutionary processes, or the presence of genetic breaks or gradients (Holderegger & Wagner, 2008; Holt & Gaines, 1992; Manel et al., 2003; Storfer et al., 2007). Understanding these fine‐scale processes allows us to better understand the underlying factors that shape biodiversity patterns and drive the population dynamics of species within an area.

For management or conservation plans, genetic information is typically incorporated at larger spatial scales (regional to global scales; Doerr et al., 2011), while fine‐scale genetic information is often unaccounted for. Several studies have, however, alluded to the importance of considering various spatial and temporal scales for effective conservation (e.g., Borda‐de‐Água, 2019; Campagnaro et al., 2017; Collins et al., 2019; Garnett et al., 2018; Henle et al., 2014; Winberg et al., 2007). Importantly, fine‐scale population genetic analyses are used as a fundamental basis to evaluate how, or even whether, local heterogeneous landscapes impose selection pressures that drive adaptation at the microhabitat scale.

This study focuses on the fine‐scale spatial genetic structure (SGS) in the collembolan *Cryptopygus antarcticus travei* Deharveng, 1981 on the sub‐Antarctic Marion Island (46°54′S, 37°45′E). Collembola (springtails) are important components of the assemblages of soil biota and occupy a myriad of niches across broad biogeographic regions (Cicconardi et al., 2013; Deharveng, 1981; Hopkin, 1997). Their strong dependence on soil ecosystems, which has driven unique biochemical and physiological adaptations, makes them particularly useful as model organisms for understanding adaptation to environmental conditions (Deharveng et al., 2008; Hopkin, 1997; Hoskins et al., 2015; Sinclair et al., 2003; Zhang et al., 2019).

Marion Island lies approximately 2200 km south‐east of the African continent and, together with Prince Edward Island, forms the Prince Edward Islands Archipelago (PEIA). These two islands represent the peaks of coalescing shield volcanoes (Rudolph et al., 2021), while the landscape is shaped by repeated large‐scale volcanic and glacial events (see Rudolph et al., 2022). Due to its location close to the Antarctic Convergence (also known as the Antarctic Polar Front), the PEIA has experienced considerable climatic changes on both geological and near‐contemporary temporal scales (Chown & Froneman, 2008; Hodgson et al., 2014; Le Roux & McGeoch, 2008; McDougall et al., 2001; Nel et al., 2020, 2023; Rouault et al., 2005; Rudolph et al., 2020; Smith, 2002). Together, these environmental changes have triggered landscape responses that have, in turn, modified the ecosystems of the islands. This makes the islands of the PEIA natural laboratories to investigate the effect of landscape complexity on the spatial genetic structure of their fauna. In particular, species such as *C. a. travei*, which are minute in size (1–2 mm; Deharveng, 1981), would be expected to be strongly impacted by small‐scale landscape complexity, given previous evidence of fine‐scale structuring due to landscape changes (Carapelli, Convey, et al., 2017; Jansen van Vuuren et al., 2018; von Saltzwedel et al., 2016).

Studies across Marion Island have confirmed the influence of landscape heterogeneity in shaping spatial genetic patterns of a wide variety of organisms (Chau et al., 2019; Grobler et al., 2006, 2011; McGaughran, Convey, et al., 2010; McGaughran, Stevens, & Holland, 2010; Mortimer et al., 2012; Mortimer & Jansen van Vuuren, 2007; Myburgh et al., 2007). Only one study on Marion Island, however, has implemented a fine‐scale sampling approach at a scale of <1 km, focusing on a keystone plant species (Born et al., 2012).

For the current study, a comprehensive spatial dataset was compiled, including geological and topographical data, across the sampled areas. This was done by developing a high‐resolution terrain resistance map and by incorporating novel data on surface geology to delineate the lava flow boundaries (see Rudolph et al., 2020). This map was used to explicitly test the effect of known geological and landscape features (see Rudolph et al., 2020, 2021, 2022) on the genetic population structure of *C. a. travei*. We hypothesise that the heterogeneous landscape created by distinct lava formations which influences the physicochemical properties of the soil and thus vegetation biomes, drive genetic patterns that may also reflect microhabitat adaptations and preferences.

## 2 METHODS

### 2.1 Study area and sample collection

**FIGURE 1:**
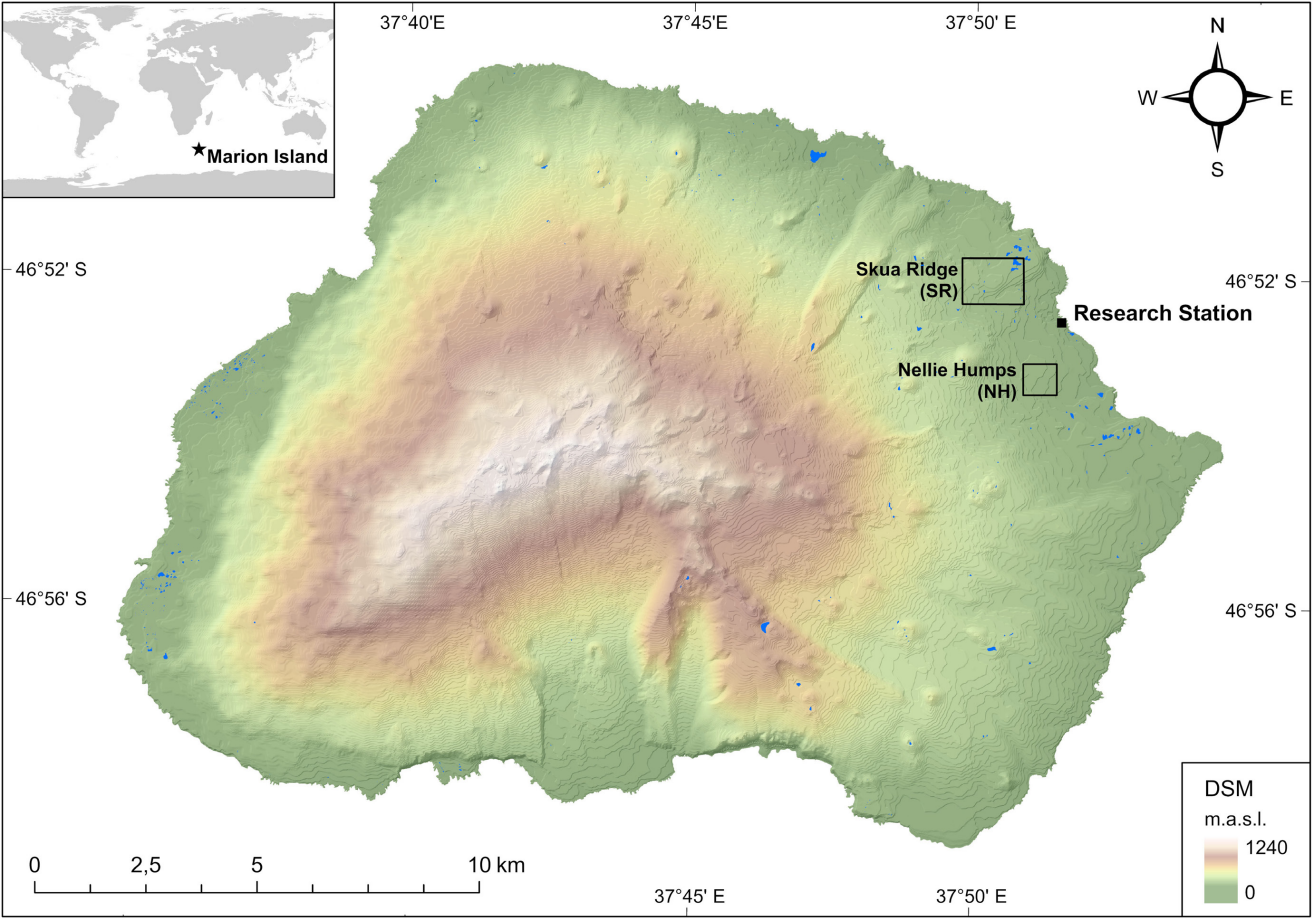
Location map of the Skua Ridge (SR) and Nellie Humps (NH) sampling sites on Marion Island. A DSM draped over a hillshade model (both at 1‐m resolution) was used to depict the topography of Marion Island. The inset map shows the geographic location of Marion Island.

**FIGURE 2:**
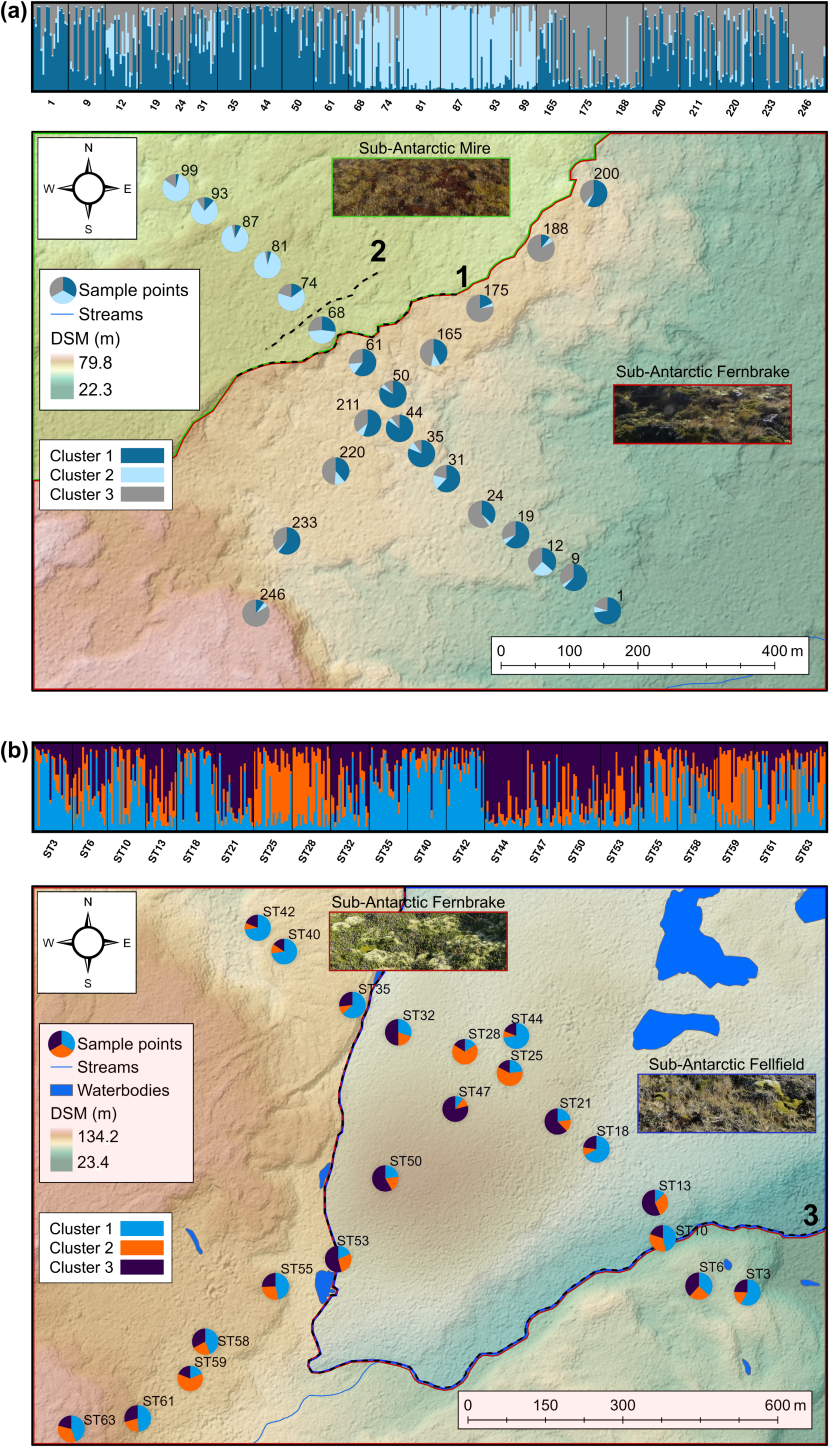
Bayesian clustering analysis illustrating spatial genetic structure of *Cryptopygus antarcticus travei* across (a) Nellie Humps (NH) and (b) Skua Ridge (SR). Barplots represent the probability of membership of each individual to a cluster. Membership probabilities of the individuals assigned to each cluster are summarised by the pie charts, with their placements corresponding to the sampling localities and plotted on a DSM draped over a hillshade model with a 1‐m resolution. Major topographical barriers are represented by the dotted lines on the hillshade models and the differences in vegetation complexes are shown by the insets. Further details on the geological barriers for SR are visualised in Figure S1. Site numbering corresponds to the numbering scheme given during the sampling.

Transect sampling was conducted on eastern Marion Island in two sampling areas, namely Nellie Humps (NH; 46°53′13.95″ S, 37°51′8.72″ E) and Skua Ridge (SR; 46°52′7.43″ S, 37°50′10.68″ E) (Figure 1); these sites are approximately 2.4 km apart. The transects were positioned in areas with high levels of habitat heterogeneity, consisting of grey and black lava formations of varying geological ages, rivers, footpaths and various vegetation biomes. Each sampling area encompassed two transects approximately 1 km in length, which were positioned perpendicular to one another to form a cross shape. The NH site included 24 sampling points separated from each other by geographic distances ranging from 30 to 120 m, while the SR site comprised 21 sampling points separated by distances ranging from 60 to 190 m (see Figure 2a,b).

*Cryptopygus a. travei* specimens were collected by extracting them from sampled moss and/or ferns (approximately 10 cm^3^) using Berlese–Tullgren funnels (Berlese, 1905; Tullgren, 1918). Twenty individuals of *C. a. travei* from each sampling point were identified and sorted using a compound light microscope and stored in absolute ethanol (Merck, South Africa).

### 2.2 Microsatellite genotyping

Whole genomic DNA was extracted using the DNeasy® Blood and Tissue Kit (Qiagen^®^, Hilden, Germany), according to the manufacturer's recommendations, with minor modifications that included a longer digestion step (overnight, but not more than 20 h) and a reduced final elution volume (75 μL instead of 100 μL). Microsatellite loci were amplified in multiplex reactions (seven multiplex sets based on the criteria described by Rastorgueff et al., 2016) using 21 highly variable and species‐specific markers (Rastorgueff et al., 2016) and fragment analysis was performed on a MultiGene™ OptiMax Thermal Cycler (Applied Biosystems). Cleaned PCR amplicons were sized using the ABI Prism^®^ 3500XL Genetic Analyser (Applied Biosystems, Foster City, California, USA). Alleles were analysed, scored and binned manually using the Geneious v8.1.5 microsatellite plugin 1.4 (Kearse et al., 2012).

To ensure standardised scoring, the scoring criteria developed by Rastorgueff et al. (2016) were followed. To guarantee genotyping quality, the data were tested for null alleles and allelic dropout using MICRO‐CHECKER (Van Oosterhout et al., 2004). Departures from Hardy–Weinberg equilibrium (HWE) were determined per locus using GenAlEx v6.502 (Peakall & Smouse, 2012) and per sampling point using GENEPOP v1.2 (Raymond & Rousset, 1995), assuming the alternative hypothesis of a deficit of heterozygotes (MICRO‐CHECKER identified an excess of homozygotes within the dataset) with the Markov Chain parameters set to the program's default settings (dememorisation: 10,000; batches: 20; and iterations per batch: 5000).

### 2.3 Spatial data

The spatial component used for this study was generated using the geoprocessing, data management and spatial analysis tools in ArcGIS Pro 2.4.0 (ESRI 2021). Using this geoprocessing toolbox, individual line segments were manually digitised to connect all sampling points. The Euclidean distance (i.e., straight line distance), surface distance and terrain roughness/ruggedness for each pair of sampling points were calculated to create a comprehensive spatial dataset.

### 2.4 Landscape and population genetic analyses

#### 2.4.1 Genetic diversity

A series of summary statistics were calculated for all sampling sites to assess genetic diversity within each sampling point using GenAlEx and the R package diveRsity (Keenan et al., 2013) in R v4.1.1 (R Core Team, 2017). GenAlEx was used to determine the number of alleles (*N*_A_), the number of effective alleles (*N*_E_), the observed heterozygosity (*H*_O_) and the expected heterozygosity (*H*_E_). A Wilcoxon signed‐rank test (Wilcoxon, 1945) was conducted in SPSS v27 (SPSS, Inc., Chicago, IL, USA) to assess for significant differences between *H*_O_ and *H*_E_ for each site. DiveRsity was used to calculate the inbreeding coefficients (*F*_IS_) per sampling unit with a 95% confidence interval for each metric using the *divBasic* function with a bootstrap of 2000 Monte Carlo replicates.

#### 2.4.2 Population genetic structure

The initial assessment of population structure was done through a non‐spatial Bayesian clustering analysis in STRUCTURE v2.3 (Pritchard et al., 2000). An admixture ancestry model with uncorrelated allele frequencies was employed with a batch run of *K* 1–10 for both NH and SR, using 10 iterations and 1,000,000 MCMC repetitions with a burn‐in of 10%. STRUCTURE HARVESTER (Earl & VonHoldt, 2012) was used to identify the most likely number of populations (*K*) using Δ*K* (Evanno et al., 2005) and the estimated log‐normal probability L(*K*). STRUCTURE plots were visualised using CLUMPAK (Kopelman et al., 2015). Genetic landscape interpolations were generated in Alleles in Space (AIS; Miller, 2005), which uses a triangulation‐connectivity network to assess genetic differences at the midpoints between neighbouring sampling localities, which were then overlaid onto the landscape maps for both sites.

To further evaluate population structure, a global *G*″_ST_ (given that *G*″_ST_ is the most suitable *F* statistic for microsatellites; Meirmans & Hedrick, 2011) was calculated using GenAlEx (9999 permutations and 10,000 bootstraps) for both NH and SR independently. The pairwise *G*_ST_ between sites assigned into clusters based specifically on geography for both NH and SR (see dotted lines 1 and 3 in Figure 2) was assessed to corroborate the presence and permeability of genetic discontinuities that are present across these barriers within the sampling area. This was implemented in FSTAT v2.9.4 (Goudet, 2003) and tested through 15,000 permutations. An analysis of molecular variance (AMOVA; Excoffier et al., 1992) using Arlequin (Excoffier et al., 1992) (99,000 permutations) was used to identify the partitioning of genetic variation among and within the spatial sampling points for both NH and SR.

Isolation‐by‐distance (Slatkin, 1993; Wright, 1943) was evaluated using Mantel tests (Mantel, 1967) with the R package adegenet (Jombart, 2008) based on 9999 permutations. Adegenet creates a density‐based scatterplot using a two‐dimensional kernel density estimation (kde2d) to illustrate the densities of the data points, along with discontinuities as patches within the density cloud. Nei's genetic distance (Nei, 1987) was computed in adegenet and the geographical distance matrices (Euclidean and surface distances) were used as the geographical measures. Spatial autocorrelation analyses were conducted in GenAlEx (9999 permutations and 10,000 bootstraps) to determine the distance at which relatedness breaks down for both the Euclidean and surface distance matrices, correlated to Nei's genetic distance.

#### 2.4.3 Landscape resistance

Landscape resistance for each sampling area was determined using the circuit theory to model connectivity employed by Circuitscape v4.0.5 (McRae, 2006; McRae et al., 2008) under the pairwise modelling mode; the digital surface model (DSM) of elevation and the terrain ruggedness index (TRI) (in ASCII format) was used as the raster resistance model inputs. The cumulative current maps generated for each sampling area were then visualised in QGIS v3.18 (QGIS Development Team, 2022) with an overlay of the DSMs of the sampling areas.

#### 2.4.4 Effects of landscape heterogeneity on genetic patterns using generalised linear models

To assess the influence of the landscape features on the genetic structure, several correlative models between genetic and landscape variables were built and compared in R. The genetic kinship, *F*_*ij*_ (Loiselle et al., 1995), between each pair of individuals was computed using SPAGeDi v1.5 (Hardy & Vekemans, 2002). This genetic metric was chosen as it is frequently used for populations with complex mating systems that are not in equilibrium and for datasets with low allele frequencies (Vekemans & Hardy, 2004). A comprehensive dataset between each pair of individuals was created, comprising the Euclidean distances, surface distances and resistance values based on the DSM and TRI runs obtained from Circuitscape. In addition, a binary matrix was created to measure the presence or absence of landscape barriers between each pair. A binary variable of 1 indicates the presence of a particular barrier, while a value of 0 indicates its absence. The following landscape barriers were identified for Nellie Humps: the topographical discontinuity (‘topographical_barriers’ for the model testing) between sites 61 and 68 is shown in Figure 2a (dotted line 1). Additionally, and based upon the hillshade model in Figure 2a, a cooling ridge of the lava flow between sites 68 and 74 (dotted line 2) was incorporated as a barrier in the model testing in combination with the abovementioned lava flow (referred to as ‘barriers_cooling_ridge’ for the model testing). For Skua Ridge, geological breaks between black and grey lava flows were identified between sites ST6 and ST10, sites ST32 and ST35 and sites ST53 and ST55 (Figure 2b; dotted line 3) and between two black lava flows (sites ST61 and ST63) (see Figure S1 for details on geological barriers). The model testing for the geology data for SR was done in two ways. First, by testing each lava flow independently (i.e., individuals on a specific lava flow versus every other individual on the alternative lava flows) and secondly, by combining all lava flows into a single model. In addition, the presence of the Van den Boogaard River, independent of the lava flows (between Site ST6 and ST10), was assessed as a potential barrier to gene flow, along with testing a smaller anthropogenic‐related barrier in the form of a footpath between sites ST53 and ST55 (see Figure S1 for details).

Collinearity between explanatory variables was assessed using the *pairs* function in R and using Spearman's correlation (*r*_s_). Strong correlations were those with *r*_s_ > 0.7 (Moraes et al., 2018; Zurr et al., 2009). These explanatory variables were retained and used for the following univariate generalised linear models (GLMs) in R. Using a Gaussian distribution for the response variable (*F*_ij_), correlations between the response and explanatory variables were carried out and model performance was selected using Akaike's information criterion (AIC; Akaike, 1974). Upon retaining the top univariate model with the lowest AIC (Burnham & Anderson, 2002), we then included the additive effect of the landscape features in the multivariate models. Finally, a null model representing the absence of an effect was included.

## 3 RESULTS

### 3.1 Data quality and statistical analyses

The complete datasets were assessed for null alleles and allelic dropout to ensure genotyping quality. No allelic dropout was reported across all loci for both sites, but Oosterhout frequencies (a frequency ≥ 0.05) suggested that null alleles may be present within the datasets (15 loci for NH and 13 loci for SR). The number of loci that deviated from the HWE for NH and SR was 16 and 18, respectively. However, all loci were retained to prevent information loss. Overall, the total number of individuals successfully genotyped for the NH site was 430 individuals (<10% missing data) and 411 individuals for the SR site (<3% missing data).

### 3.2 Landscape and population genetic analyses

#### 3.2.1 Genetic diversity

The levels of genetic diversity within each sampling point of the NH site are presented in Table S1, with the number of alleles (*N*_A_) ranging from 3.19 to 4.71 and the number of effective alleles (*N*_E_) ranging from 2.03 to 2.69. The observed (*H*_O_) and expected heterozygosity (*H*_E_) values ranged from 0.26 to 0.43 and 0.42 to 0.55, respectively (the Wilcoxon signed rank test revealed a significant difference between observed and expected heterozygosity, *n* = 24, *Z* = −4.290, *p* < .001). The 95% confidence intervals of the inbreeding coefficients (*F*_IS_) highlight no departure from HWE for only two of the NH localities (sites 24 and 50). For SR (Table S2), the number of alleles (*N*_A_) ranged from 3.57 to 4.71 and the number of effective alleles (*N*_E_) ranged from 2.14 to 2.46. The observed (*H*_O_) and expected heterozygosity (*H*_E_) values at SR ranged from 0.28 to 0.44 and 0.44 to 0.50, respectively (again, the Wilcoxon signed rank test revealed a significant difference between observed and expected heterozygosity, *n* = 21, *Z* = −4.021, *p* < .001). The 95% confidence intervals for the inbreeding coefficients (*F*_IS_) revealed that only one site (ST59) did not deviate from HWE.

#### 3.2.2 Population genetic structure, genetic diversity and spatial analyses

The Bayesian clustering method showed that the most probable number of clusters for both NH and SR, based on Δ*K* and L(*K*), was three, with high levels of individual variation and admixture for both sampling sites (Figure 2a,b). For NH, the genetic structure between clusters 1 and 2, at sites 61 and 68, coincides with a geological ridge as demonstrated by the hillshade model (Figure 2a; dotted line 1). For site SR, there was no distinct pattern of genetic structure corroborating with the landscape (Figure 2b). The AMOVA results for both NH and SR (Tables S3 and S4) confirm the intra‐population variation inference of a high admixture content as both sites revealed the extent of within‐individual variation (NH: 74.42% and SR: 74.35%). The global *G*″_ST_ value across all loci for NH was 0.141, indicating an overall greater genetic structure compared to SR, which was 0.068. The pairwise *G*_ST_ between clusters on either side of the geographical barriers for NH (see the dotted line 1 in Figure 2a) was calculated to be 0.670, while the pairwise *G*_ST_ between the clusters on either side of the geological barrier for SR (i.e., black lava outflow versus grey lava outflow; see dotted line 3 in Figure 2b and see also Figure S1) was calculated to be 0.5.

The correlation between genetic distance versus Euclidean distance and surface distance was positive (only the Mantel test using Euclidean distance is shown, as the correlation and significance was identical when using the surface distance matrix; NH: Figure S2; SR: Figure S3). Both analyses show a positive correlation that was significant for NH (NH: *r*_s_ = .45, *p* < .0001), indicating the presence of isolation‐by‐distance for NH, but not for SR (SR: *r*_s_ = .113, *p* > .05), which corroborates with the finding of overall weak genetic structure for SR. The spatial autocorrelation analyses illustrate a non‐significant positive correlation and relatedness up to a distance of 171.21 m for the Euclidean distance measure (Figure S4a) and 177.24 m for the surface distance matrix (Figure S4b) for NH. For SR, the relatedness was determined to break down at 196.54 m (Euclidean distance matrix; Figure S5a) and 403.07 m (surface distance matrix, Figure S5b).

**FIGURE 3:**
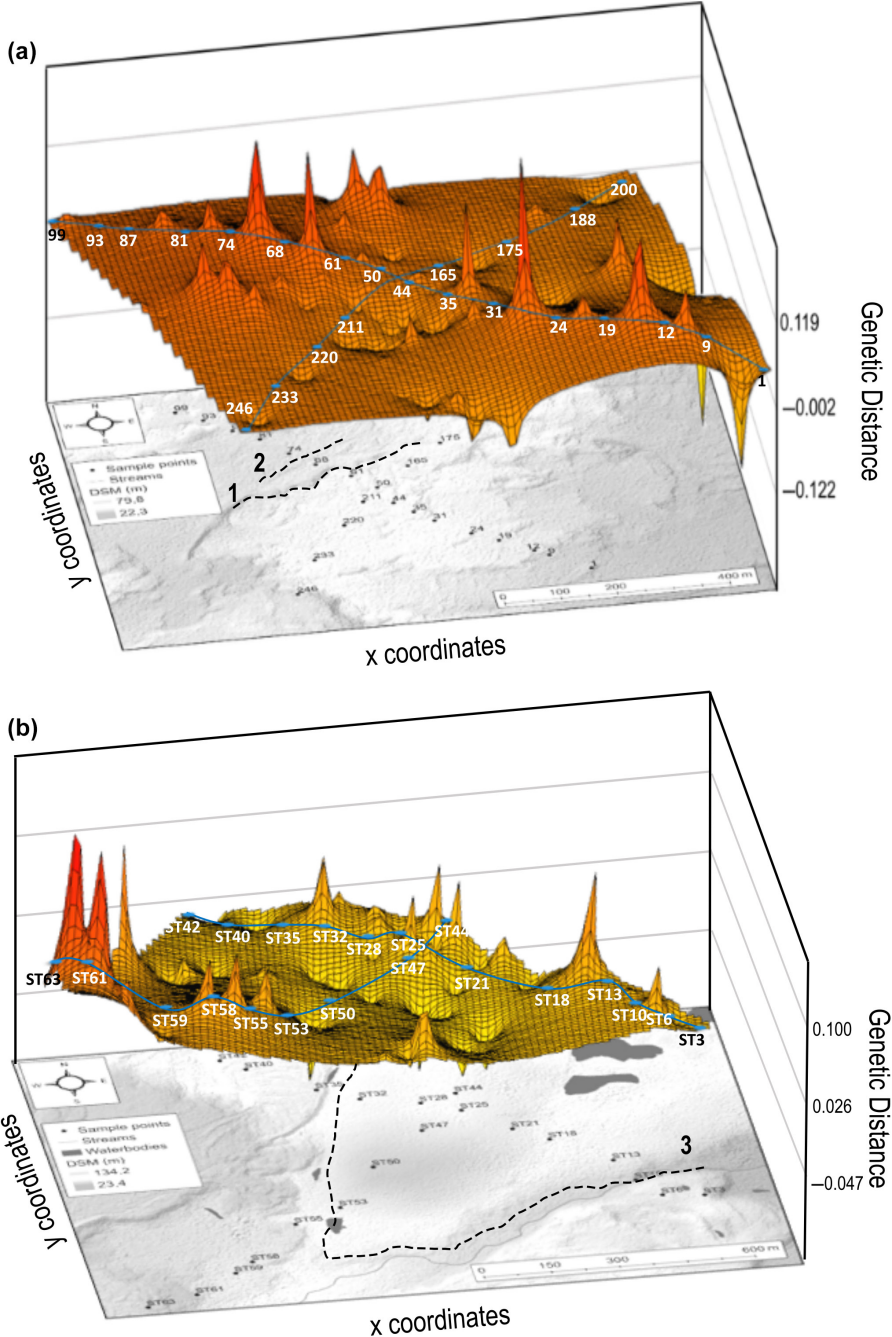
Genetic landscape interpolation plots for (a) Nellie Humps (NH) and (b) Skua Ridge (SR) to visualise the levels of genetic differentiation at the midpoints between neighbouring sites using a triangulation‐connectivity network. The genetic differentiation is visualised in the form of peaks (high level of differentiation) or troughs (low level of differentiation) and overlaid onto the hillshade model of the region, with the prominent topographical barriers represented by the dotted lines.

The genetic landscape interpolation plots for NH (Figure 3a) and SR (Figure 3b) highlight the overall elevated inter‐population genetic differences (peaks), likely owing to the high levels of variation accounted for by each site. For NH, the overall genetic differentiation has a larger range from −0.122 (troughs) to 0.119 (peaks), compared to the smaller range for SR with a minimum of −0.047 (troughs) and a maximum of 0.100 (peaks). For NH, notable higher levels of variation are seen between sites 24 and 31, sites 31 and 35, sites 61 and 68 and between sites 68 and 74, possibly due to the geological features evident in the hillshade model and confirmed by the high levels of landscape resistance between these sites (Figure S6). For SR, high levels of genetic differentiation are present between sites ST13 and ST18, ST59 and ST61, ST61 and ST63 and ST42 and ST63, which could be a result of landscape distance and resistance visualised in Figure S7.

#### 3.2.3 Testing the effects of landscape heterogeneity using GLMs

The univariate GLMs revealed that the best explanatory variable for genetic structure across both NH and SR was landscape resistance inferred from the DSMs for the sites (Table S5 for NH and Table S6 for SR). The multivariate analyses demonstrated that for NH (Table S7), the most plausible model explaining spatial structure was landscape resistance concurrently with the edges of the distinct lava flows visible in Figure 2a and as described above. For SR, the best multivariate model (Table S8) to explain genetic structure was landscape resistance in combination with the grey lava flow, which presents a barrier between sites ST6 and ST10, ST32 and ST35 and ST53 and ST55 (refer to Figure 2b and Figure S1 for details).

## 4 DISCUSSION

Knowledge of population genetic diversity and structure is important when confronting issues that lead to biodiversity loss and species extinction (Borda‐de‐Água, 2019; Collins et al., 2019; Garnett et al., 2018; Hill et al., 2018). Previous large‐scale analyses (hundreds of kilometres) using the mitochondrial COI gene and a few microsatellite markers in the Antarctic and sub‐Antarctic regions indicated that the genetic structure of terrestrial and marine organisms is likely shaped by various combinations of glaciers, glacial refugia, geographical distances and ocean depth between regions (Beet et al., 2016; Collins & Hogg, 2016; Fanciulli et al., 2001; Jansen van Vuuren et al., 2018; Lanusse et al., 2022; McGaughran et al., 2008; Mortimer et al., 2011; Strugnell et al., 2017). For sub‐Antarctic Marion Island, studies of various terrestrial taxa have revealed that genetic structuring is shaped by both historical (volcanic and glacial episodes) and contemporary climatic conditions, which have created environmental variability on both regional (island) and local scales (Chau et al., 2019; Grobler et al., 2006, 2011; McGaughran, Convey, et al., 2010; McGaughran, Stevens, & Holland, 2010; Mortimer et al., 2008, 2012; Mortimer & Jansen van Vuuren, 2007; Myburgh et al., 2007). Globally, fine‐scale analyses (tens of kilometres) have been carried out for a multitude of taxa, using both mitochondrial DNA and microsatellite markers (Beheregaray & Sunnucks, 2001; Bull et al., 2013; Dawson Pell et al., 2021; Garrick et al., 2004, 2008; Shchipanov et al., 2022; Sunnucks et al., 2006). Only one study at a fine spatial scale (~1 km) has so far been conducted on a Marion Island species and is the only one of its kind conducted in the sub‐Antarctic region. That study, which focused on the cushion plant *Azorella selago*, identified exceptionally complex patterns at a local scale, which would have gone un‐noticed at a larger spatial scale (Born et al., 2012). The present study of *Cryptopygus antarcticus travei* adds valuable new information to better understand the effects of local‐scale landscape heterogeneity on the population genetic structure of the island's unique biota.

Populations of *C. a. travei* on Marion Island harbour high levels of genetic diversity, consistent with previous studies that have also reported high levels of diversity in other species of Collembola (Carapelli, Convey, et al., 2017; Carapelli, Leo, & Frati, 2017; Collins et al., 2019; McGaughran et al., 2008, 2011, 2019; McGaughran, Torricelli, et al., 2010; Rastorgueff et al., 2016; Torricelli et al., 2010; van der Wurff et al., 2005). All of these studies, however, have been conducted at large spatial scales (i.e., north‐west Europe, regions of Antarctica and across sub‐Antarctic and/or Antarctic islands). Therefore, the high levels of genetic diversity found in this study (based on expected heterozygosity) at a fine spatial scale provide an important and novel observation for the group.

For all sampling points from both Nellie Humps and Skua Ridge, significant differences between *H*_O_ and *H*_E_ were observed, with *H*_O_ consistently being significantly lower than *H*_E_, suggesting departures from HWE (assumption of panmixia) resulting from genetic structure at small spatial scales. Furthermore, the percentage of variation accounted for by the ‘within individuals’ component of the AMOVA tests show that intra‐population variation is exceptionally high.

Bayesian clustering analysis suggests a complex spatial genetic pattern for Nellie Humps and, when superimposed on the digital surface and hillshade models of the landscape (see Figure 2a), revealed that a major break in slope/landscape feature broadly coincides with the genetic discontinuity across the sampling transect between sites 61 and 68 (see Figure 2a; dotted line 1). This is due to the formation of black lava outflows of different ages, whereby the edge of the black lava flow intersects the sampling area between sites 61 and 68. As a result, floral and faunal recolonisation has led to genetically distinct populations. The higher levels of genetic diversity for sites from cluster 1 and 3 compared to cluster 2 potentially validate the occurrence of a more recent lava flow (at sites 68–87), which led to population bottlenecks and thus founding events post‐lava formation. Although significant genetic structure was identified between the clusters across the break, the individual membership probabilities illustrate that gene flow was found across these areas, which is evident from the fact that even though most individuals from a particular area were assigned to a particular cluster, most also had ancestry components from other clusters. The Bayesian clustering analyses for Skua Ridge demonstrated no clear pattern of genetic structuring linked to landscape heterogeneity, with most sampling localities comprising admixed individuals with equal cluster memberships. However, when comparing sites on either side of the grey lava outflow edge (see dotted line 3 in Figure 2b), significant genetic structure is evident which corroborates the evidence from GLMs that the grey lava flow is the most probable model driving genetic structure.

Several previous studies have confirmed that geographical distances are not the only factors affecting gene flow, but that environmental/landscape factors play a crucial role (Jiang et al., 2019; Sexton et al., 2013; Shafer & Wolf, 2013; Wang & Bradburd, 2014). The GLMs illustrated that the plausible model for shaping the structure for Nellie Humps is landscape resistance in combination with the topographical barriers. For Skua Ridge, the GLMs highlight the specific influence of terrain resistance in concert with the geology (i.e., grey lava outflows) and potentially the associated vegetation differences on genetic structure.

Based on ground observations, the vegetation biomes and community complexes across the Nellie Humps sampling transect comprises the sub‐Antarctic Mire Vegetation and sub‐Antarctic Fernbrake Vegetation biomes (see Mucina & Rutherford, 2006 for details). Fine‐scale differences in the vegetation types at each sampling site are, however, evident. Briefly, the southeastern area of the sampling site is dominated by sub‐Antarctic Fernbrake Vegetation (predominantly *Blechnum penna‐marina* and *Azorella selago*, with exposed rocky outcrops; see vegetation insets in Figure 2a). The northwestern section of the sampling area is dominated by sub‐Antarctic Mire Vegetation with the occasional instance of *B. penna‐marina* (i.e., fernbrakes), which is common on younger black lava landforms (Mucina & Rutherford, 2006). For Skua Ridge, the grey lava is dominated by the sub‐Antarctic Fellfield Vegetation biome comprising mostly of *Agrostis magellanica*, *A. selago*, *Racomitrium lanuginosum* and sparse *B. penna‐marina*, while the black lava flows are characterised by Fernbrake Vegetation dominated by *B. penna‐marina*, *Poa cookii* and *Acaena magellanica* (see vegetation insets in Figure 2b). The geological features created by volcanic eruptions influence the physicochemical properties of the soil, therefore, the mineral and moisture contents of the soil differ significantly per vegetation biome across Marion Island (Mucina & Rutherford, 2006; Neall, 2009). Considering the variability of soil chemistry and properties across the region, which are driven by different geological formations, drastic changes in vegetation complexes are noticeable. Microhabitat variability has been documented whereby indigenous collembolans prefer drier soils with low organic carbon content and species richness has been documented to be greater in cold fellfield (e.g., high‐altitude) areas (Gabriel et al., 2001). Gabriel et al. (2001) reported that springtail assemblages differ according to soil structure and chemistry (i.e., pH, total sodium, exchangeable sodium, total nitrogen, total phosphorous, phosphate, organic carbon and moisture content), as well as temperature. Springtail assemblages have broad habitat preferences (Convey et al., 1999; Hopkin, 1997), although Gabriel et al. (2001) suggested that habitat specificity does indeed exist within collembolan communities. Therefore, differentiation between springtail assemblages across NH and SR could be partly driven by the combination of historical (geological) events and contemporary vegetation differences linked to microhabitat availability and adaptive ability.

Despite the absence of absolute dispersal barriers that would result in panmixia across the sampling area, the datasets incorporating a comprehensive spatial component and high‐resolution resistance maps showed that fine scale spatial patterns were surprisingly complex and more so than had previously been documented at large spatial scales on Marion Island (Chau et al., 2019; Grobler et al., 2006, 2011; McGaughran, Convey, et al., 2010; McGaughran, Stevens, & Holland, 2010; Mortimer et al., 2008, 2012; Mortimer & Jansen van Vuuren, 2007; Myburgh et al., 2007). Our results illustrate that, on a fine spatial scale, the island's geodiversity shapes landscape complexity and, therefore, the spatial distribution of vegetation. In combination, these factors strongly influence the spatial patterns and levels of genetic diversity of *C. a. travei* on Marion Island.

This study illustrates that the genetic patterns of *C. a. travei* on sub‐Antarctic Marion Island are considerably more structured at fine spatial scales than anticipated on the basis of the fact that habitats on Marion Island superficially appear to be continuous and that Collembola harbour high levels of genetic diversity, with evidence of genetic structuring documented at fine spatial scales (Carapelli, Convey, et al., 2017; Carapelli, Leo, & Frati, 2017; Collins et al., 2019; Jansen van Vuuren et al., 2018; McGaughran et al., 2008, 2011, 2019; McGaughran, Torricelli, et al., 2010; Rastorgueff et al., 2016; Torricelli et al., 2010; van der Wurff et al., 2005; von Saltzwedel et al., 2016). This information can be used to highlight the importance of fine‐scale spatial sampling as an essential tool in uncovering spatial patterns in genetic diversity and, potentially, local adaptations. Fine‐scale research, such as that described here, should not be overlooked in conservation biology (Borda‐de‐Água, 2019; Campagnaro et al., 2017; Collins et al., 2019; Garnett et al., 2018; Hill et al., 2018), since the levels of structure vary significantly between spatial scales. Fine‐scale work can be used to evaluate the selective pressures imposed by the local heterogeneous landscape on individuals. Using the pristine environment of Marion Island as a natural laboratory, adaptations can be assessed to the complex heterogeneous landscape of the island, which poses challenges to the survival of organisms. The rapidly changing environment, exacerbated by the rapid acceleration of climate change, compels organisms to develop mechanisms to adapt to their local habitat (Chevin et al., 2010; Visser, 2008). This study shows that populations are faced with the challenge of adapting to a changing environment and that, on Marion Island, geodiversity plays an important role in shaping biodiversity and genetic diversity. The fine‐scale genetic differences identified here among local populations on Marion Island highlight the critical role that landscape features play in maintaining genetic diversity, even on small spatial scales. The maintenance of such diversity within natural populations will be critical when faced with the challenges of a changing environment and needs to be considered as part of conservation management planning.

## AUTHOR CONTRIBUTIONS

**Daniela Marques Monsanto:** Conceptualization (equal); data curation (equal); formal analysis (lead); investigation (lead); methodology (equal); visualization (equal); writing – original draft (lead); writing – review and editing (equal). **David William Hedding:** Data curation (equal); funding acquisition (supporting); methodology (equal); visualization (equal); writing – review and editing (equal). **Sandra Durand:** Data curation (equal); methodology (equal); writing – review and editing (equal). **Shilpa Pradeep Parbhu:** Methodology (equal); writing – review and editing (equal). **Matthew Grant Adair:** Methodology (equal); visualization (equal); writing – review and editing (equal). **Arsalan Emami‐Khoyi:** Methodology (equal); supervision (equal); writing – review and editing (equal). **Peter Rodja Teske:** Supervision (equal); writing – review and editing (equal). **Bettine Jansen van Vuuren:** Conceptualization (equal); funding acquisition (lead); supervision (equal); writing – review and editing (equal).

## FUNDING INFORMATION

Funding was obtained by the National Research Foundation (NRF) Competitive Programme for Rated Researchers granted to B.J.v.V. (NRF CPRR grant number: 138016) and the NRF South African National Antarctic Programme (SANAP) granted to D.W.H. (NRF SANAP grant number: 129235).

## CONFLICT OF INTEREST STATEMENT

The authors declare that there are no conflicts of interest.

## Supporting information


Data S1.

## Data Availability

The data that support the findings of this study can be downloaded from Dryad: https://datadryad.org/stash/share/q1‐UPR1YnveWzAj4‐kPcMGN5wFBgSxGEkB7JRfbm0rU; DOI: https://doi.org/10.5061/dryad.mkkwh715s.
