# Supplementary material for: The effect of terrain on the fine‐scale genetic diversity of sub‐Antarctic Collembola: A landscape genetics approach
Source: Ecol Evol. 2024 Jun 18;14(6):e11519. doi: 10.1002/ece3.11519 (PMC11183960; doi:10.1002/ece3.11519)
Supplement: Supplementary file 1 — Data S1. [file ECE3-14-e11519-s001.docx]

**The effect of terrain on the fine-scale genetic diversity of sub-Antarctic Collembola: A landscape genetics approach**

**Data accessibility statement**

The data that support the findings of this study can be downloaded from Dryad: <https://datadryad.org/stash/share/q1-UPR1YnveWzAj4-kPcMGN5wFBgSxGEkB7JRfbm0rU>; DOI: 10.5061/dryad.mkkwh715s

**Supplementary Information**


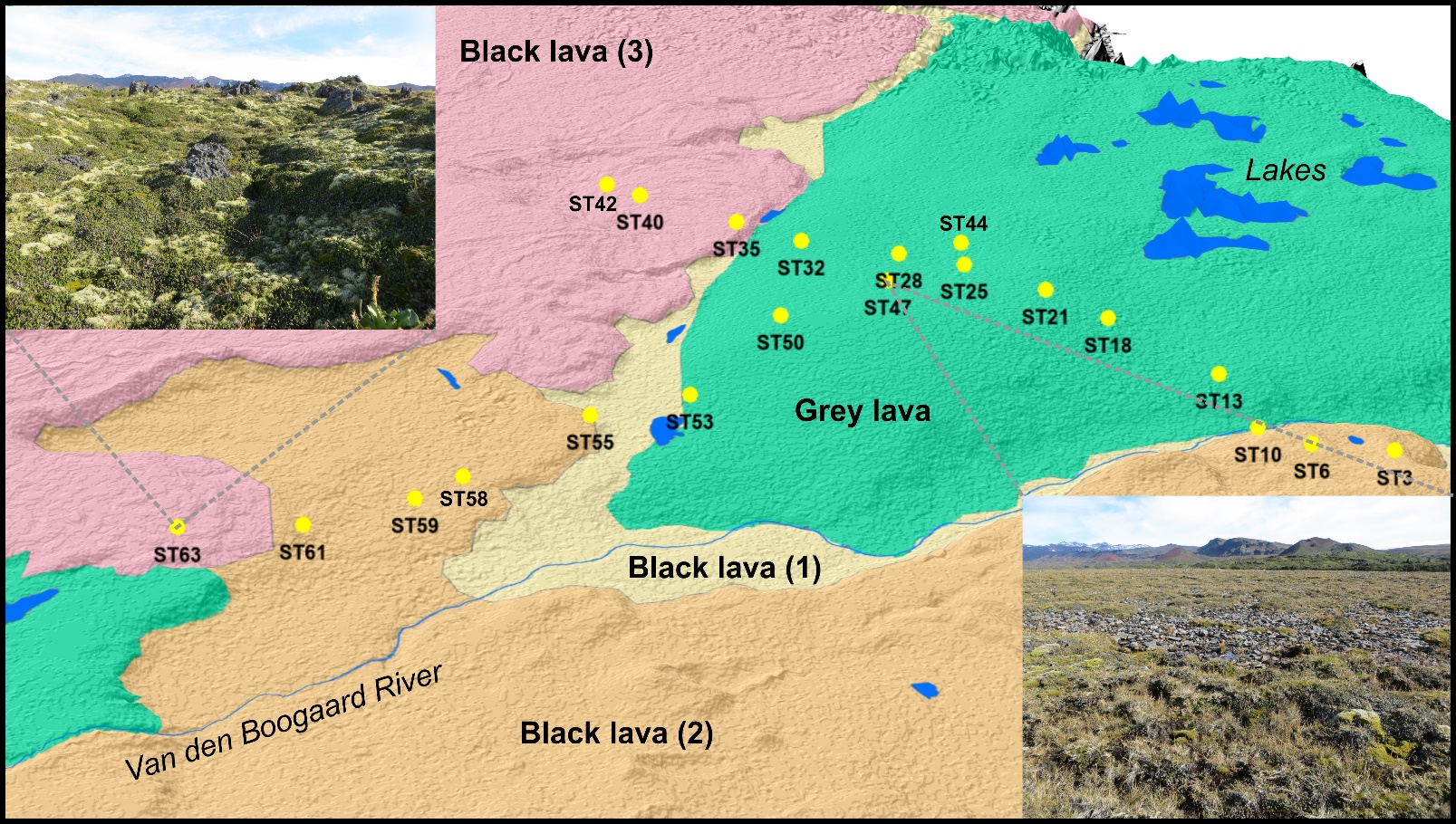


**Fig. S1** Oblique aerial view looking north over the Skua Ridge site, highlighting the geological units, the waterbodies, and the footpath (grey dotted line between sites ST53 and ST55) in the area. The insets are photographs showing the differences in vegetation across the site with the black lava outflows being dominated by the sub-Antarctic Fernbrake Vegetation biome while the grey lava is dominated by the sub-Antarctic Fellfield Vegetation biome (see Mucina and Rutherford 2006 for vegetation biomes)


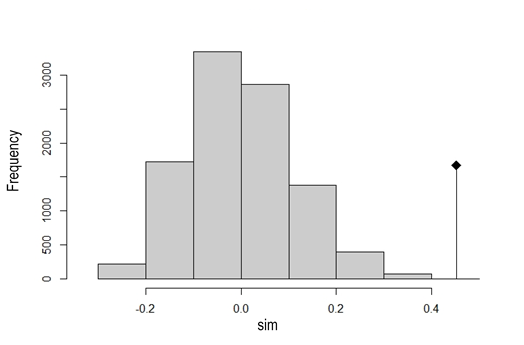

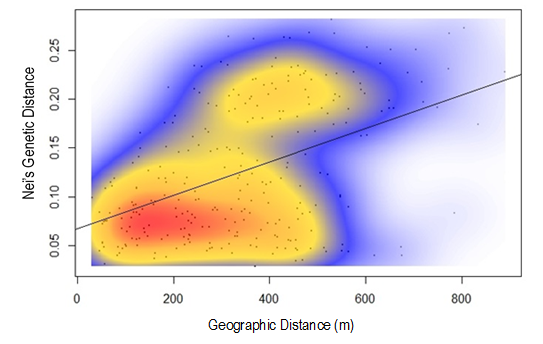


**R_s_ = 0.45; *p* < 0.0001**

a)

b)

**Fig. S2** Isolation by distance analysis for genetic distance vs Euclidean distance for the Nellie Humps sampling area. Histogram (a) of isolation by distance simulation represents the permuted value under the assumption that spatial structure is absent. The observed significant correlation between the genetic and geographical distance is overlaid and represented as a diamond. The density-based scatterplot (b) using the two-dimensional kernel density estimation (kde2d) illustrates the densities of the datapoints along with discontinuities best illustrated by patches in the density clouds. Genetic versus surface distance plot is not shown as the correlation and significance was identical to the genetic versus Euclidean distance analysis


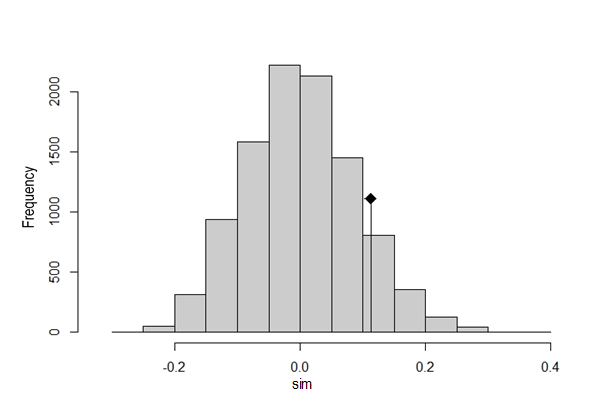

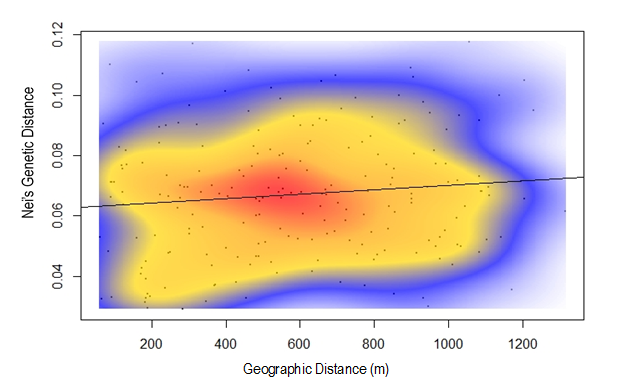


**R_s_ = 0.113; *p* > 0.05**

a)

b)

**Fig. S3** Isolation by distance analysis for genetic distance vs Euclidean distance for Skua Ridge. Histogram (a) of isolation by distance simulation represents the permuted value under the assumption that spatial structure is absent. The observed significant correlation between the genetic and geographical distance is overlaid and represented as a diamond. The density-based scatterplot (b) using the two-dimensional kernel density estimation (kde2d) illustrates the densities of the datapoints along with discontinuities best illustrated by patches in the density clouds. Genetic versus surface distance plot is not shown as the correlation and significance was identical to the genetic versus Euclidean distance analysis


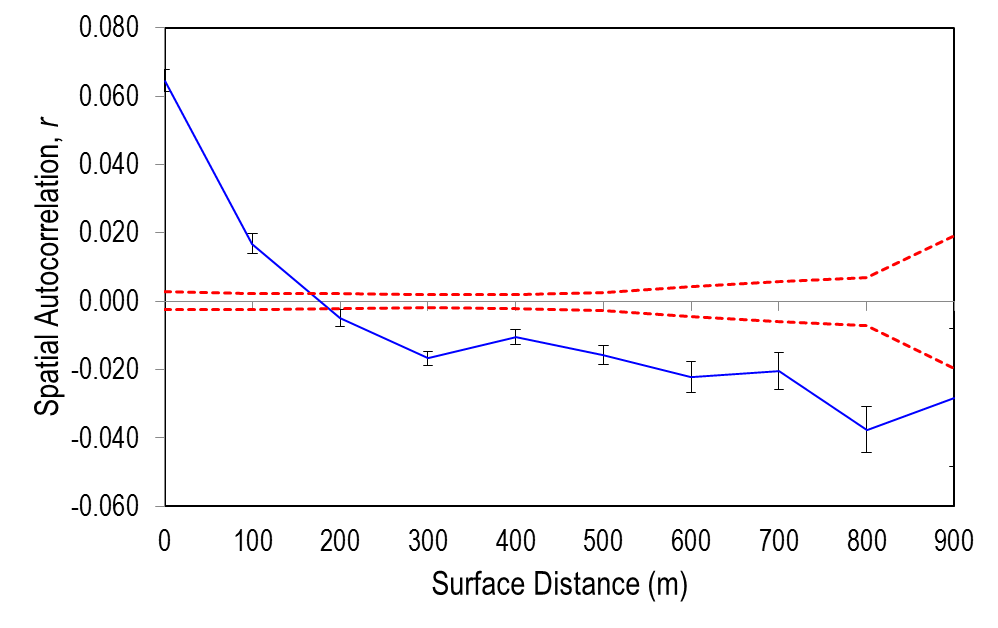

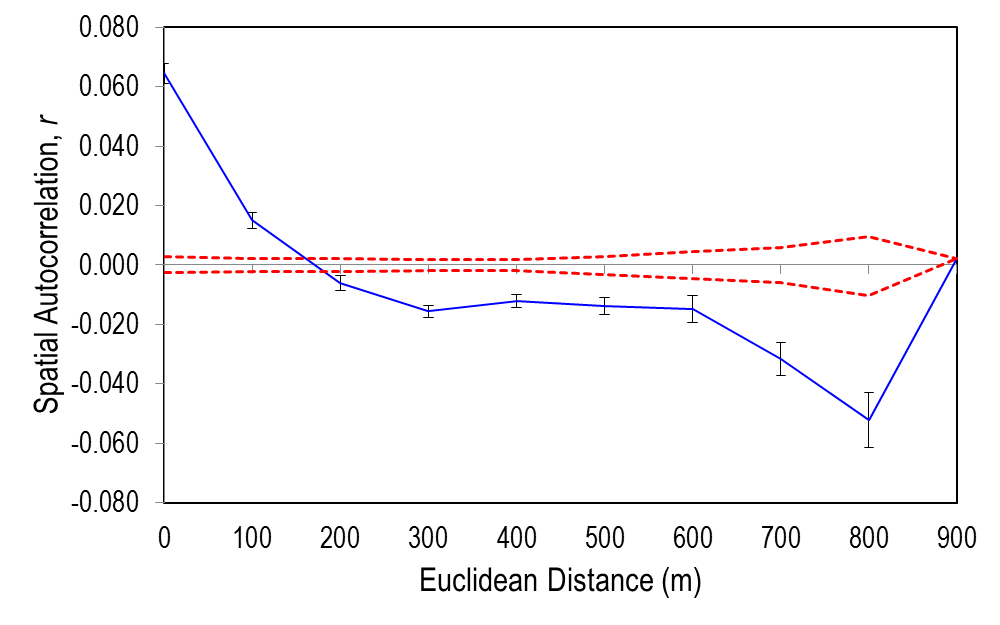


Intercept = 171.21 m

Intercept = 177.24 m

a)

b)

**Fig. S4** Spatial autocorrelation analysis for Nellie Humps using a) the Euclidean distance matrix, and b) the surface distance matrix. The correlations between the distance measures and genetic distance are shown by the spatial autocorrelation index (*r*, blue line). The red upper and lower bounds represent the 95% confidence intervals. If *r* is situated outside this 95% CI, spatial autocorrelation is considered significant (*p* < 0.05)


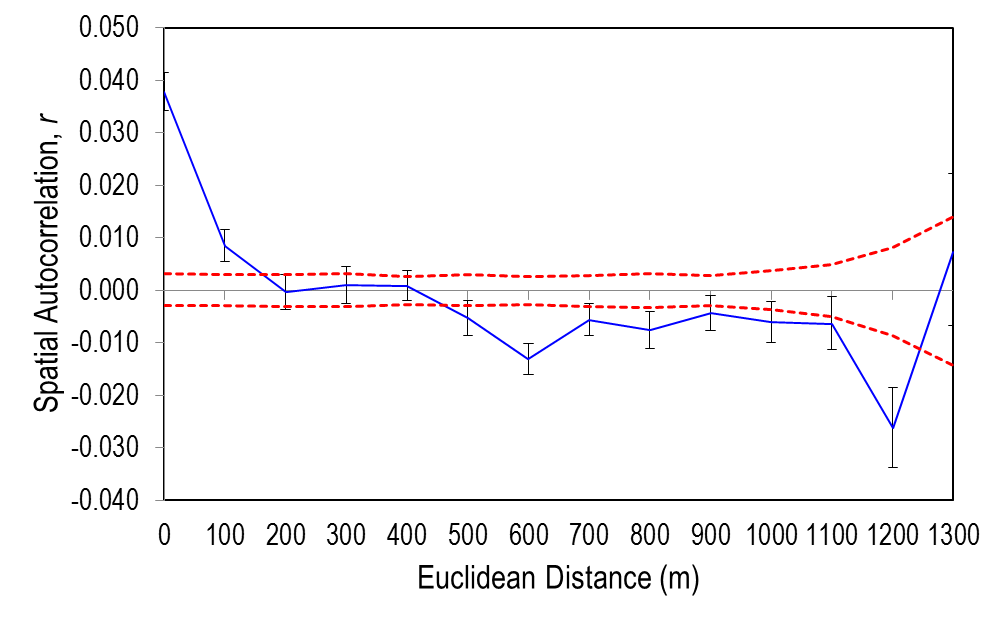

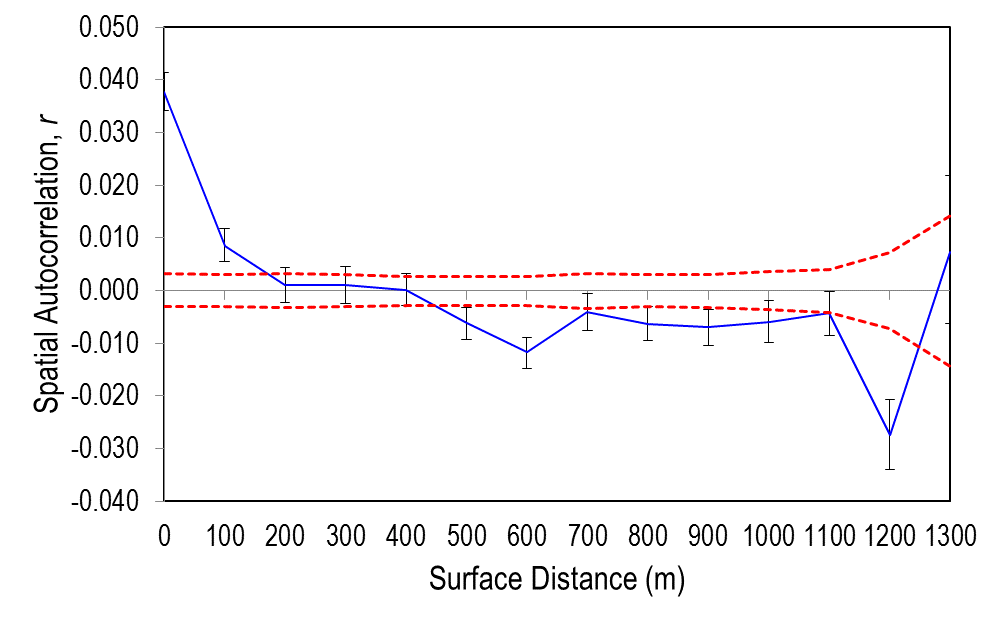


Intercept = 196.54 m

Intercept = 403.07 m

a)

b)

**Fig. S5** Spatial autocorrelation analysis for Skua Ridge using a) the Euclidean distance matrix, and b) the surface distance matrix. The correlations between the distance measures and genetic distance are shown by the spatial autocorrelation index (*r*, blue line). The red upper and lower bounds represent the 95% confidence intervals. If *r* is situated outside this 95% CI, spatial autocorrelation is considered significant (*p* < 0.05)

**
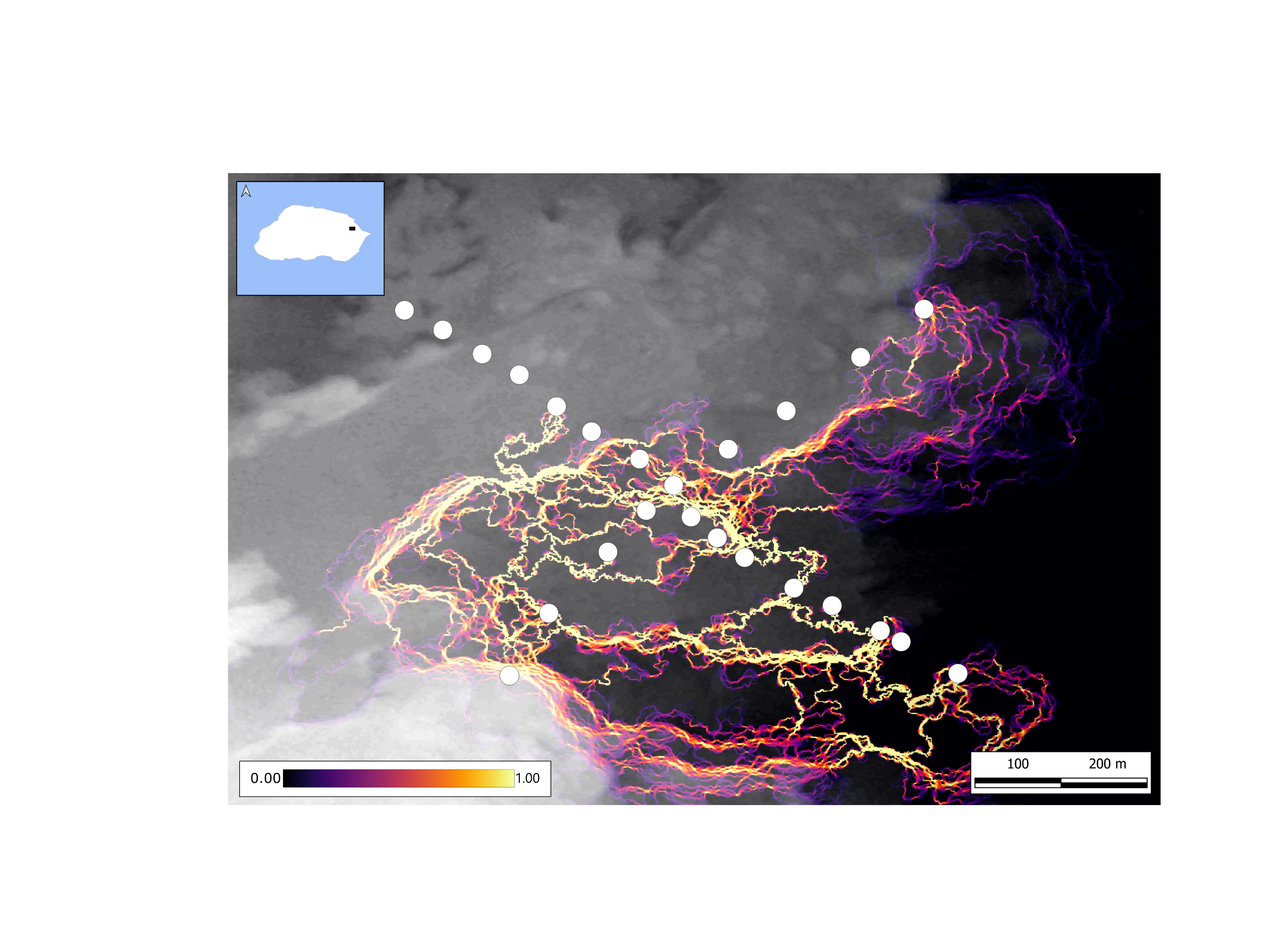
***Landscape resistance*

**Fig. S6** Cumulative current map obtained using the circuit theory for Nellie Humps depicting landscape resistance between sampling points, with an overlay of the digital surface model (grey shaded areas)

**
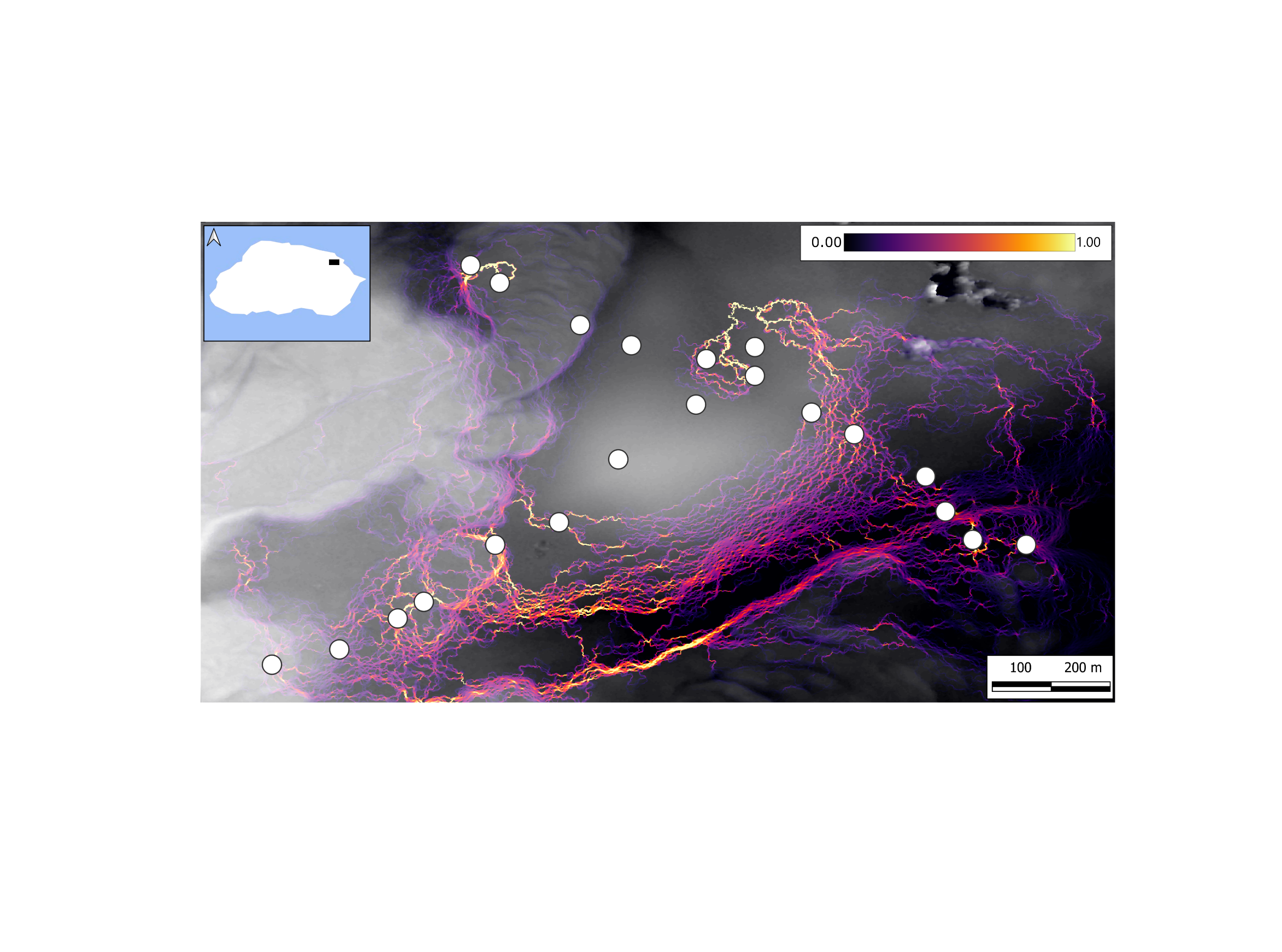
**

**Fig. S7** Cumulative current map obtained using the circuit theory for Skua Ridge depicting landscape resistance between sampling points, with an overlay of the digital surface model (grey shaded areas)

*Genetic diversity*

**Table S1** Summary statistics calculated for all spatial sampling units included in the Nellie Humps sampling area. The number of individuals per site (*N*), the number of alleles (*Na*), the number of effective alleles (*Ne*), the observed heterozygosity (*Ho*), the expected heterozygosity (*He*), and inbreeding coefficients (*F*_IS_) and the 95% confidence intervals are shown per sampling unit, as well as the means for all sites

| **Pop** | **N** | ***Na*** | ***Ne*** | ***Ho*** | ***He*** | ***F*_IS_** | ***F*_IS_ (lower)** | ***F*_IS_ (upper)** |  |
| --- | --- | --- | --- | --- | --- | --- | --- | --- | --- |
| 1 | 19 | 3.91 | 2.12 | 0.32 | 0.43 | 0.24 | 0.12 | 0.36 |  |
| 9 | 20 | 4.38 | 2.38 | 0.41 | 0.48 | 0.14 | 0.03 | 0.25 |  |
| 12 | 18 | 4.71 | 2.69 | 0.42 | 0.55 | 0.24 | 0.10 | 0.39 |  |
| 19 | 19 | 3.81 | 2.24 | 0.34 | 0.46 | 0.25 | 0.09 | 0.40 |  |
| 24 | 9 | 3.38 | 2.52 | 0.36 | 0.44 | 0.18 | -0.06 | 0.46 |  |
| 31 | 15 | 3.71 | 2.45 | 0.39 | 0.50 | 0.22 | 0.07 | 0.35 |  |
| 35 | 18 | 4.48 | 2.21 | 0.36 | 0.48 | 0.24 | 0.10 | 0.37 |  |
| 44 | 17 | 3.52 | 2.03 | 0.34 | 0.42 | 0.20 | 0.06 | 0.35 |  |
| 50 | 17 | 4.05 | 2.26 | 0.43 | 0.47 | 0.08 | -0.03 | 0.18 |  |
| 61 | 19 | 4.19 | 2.30 | 0.39 | 0.50 | 0.22 | 0.10 | 0.33 |  |
| 68 | 13 | 3.52 | 2.37 | 0.36 | 0.51 | 0.29 | 0.09 | 0.47 |  |
| 74 | 17 | 3.62 | 2.33 | 0.26 | 0.48 | 0.45 | 0.31 | 0.56 |  |
| 81 | 20 | 4.00 | 2.31 | 0.32 | 0.52 | 0.38 | 0.25 | 0.48 |  |
| 87 | 20 | 4.33 | 2.46 | 0.30 | 0.50 | 0.40 | 0.30 | 0.48 |  |
| 93 | 20 | 4.33 | 2.31 | 0.38 | 0.48 | 0.21 | 0.10 | 0.31 |  |
| 99 | 12 | 3.19 | 2.23 | 0.34 | 0.47 | 0.27 | 0.10 | 0.41 |  |
| 165 | 18 | 3.81 | 2.19 | 0.35 | 0.45 | 0.21 | 0.04 | 0.35 |  |
| 175 | 20 | 4.29 | 2.40 | 0.36 | 0.48 | 0.25 | 0.12 | 0.37 |  |
| 188 | 20 | 3.91 | 2.26 | 0.26 | 0.48 | 0.47 | 0.33 | 0.59 |  |
| 200 | 20 | 3.91 | 2.29 | 0.37 | 0.46 | 0.19 | 0.07 | 0.30 |  |
| 211 | 20 | 4.00 | 2.30 | 0.30 | 0.46 | 0.35 | 0.21 | 0.50 |  |
| 220 | 20 | 4.57 | 2.36 | 0.43 | 0.48 | 0.10 | 0.02 | 0.17 |  |
| 233 | 19 | 4.10 | 2.26 | 0.34 | 0.47 | 0.28 | 0.18 | 0.38 |  |
| 246 | 20 | 4.38 | 2.61 | 0.40 | 0.50 | 0.20 | 0.07 | 0.32 |  |
| Mean | 17.92 | 4.00 | 2.33 | 0.36 | 0.48 | 0.25 | - | - |  |

**Table S2** Summary statistics calculated for all spatial sampling units included in the Skua Ridge sampling area. The number of individuals per site (*N*), the number of alleles (*Na*), the number of effective alleles (*Ne*), the observed heterozygosity (*Ho*), the expected heterozygosity (*He*), and inbreeding coefficients (*F*_IS_) and the 95% confidence intervals are shown per sampling unit, as well as the means for all sites

| **Pop** | **N** | ***Na*** | ***Ne*** | ***Ho*** | ***He*** | ***F*_IS_** | ***F*_IS_ (lower)** | ***F*_IS_ (upper)** |  |
| --- | --- | --- | --- | --- | --- | --- | --- | --- | --- |
| ST3 | 20 | 4.43 | 2.42 | 0.36 | 0.46 | 0.22 | 0.07 | 0.35 |  |
| ST6 | 18 | 4.43 | 2.35 | 0.35 | 0.50 | 0.29 | 0.17 | 0.39 |  |
| ST10 | 20 | 3.91 | 2.24 | 0.30 | 0.45 | 0.33 | 0.21 | 0.45 |  |
| ST13 | 16 | 4.24 | 2.46 | 0.31 | 0.47 | 0.34 | 0.23 | 0.44 |  |
| ST18 | 20 | 4.19 | 2.34 | 0.32 | 0.47 | 0.30 | 0.17 | 0.43 |  |
| ST21 | 20 | 4.24 | 2.33 | 0.35 | 0.45 | 0.21 | 0.10 | 0.31 |  |
| ST25 | 20 | 4.19 | 2.37 | 0.44 | 0.50 | 0.12 | 0.01 | 0.21 |  |
| ST28 | 20 | 4.19 | 2.34 | 0.36 | 0.49 | 0.25 | 0.13 | 0.37 |  |
| ST32 | 20 | 4.48 | 2.46 | 0.41 | 0.49 | 0.17 | 0.05 | 0.28 |  |
| ST35 | 20 | 4.29 | 2.29 | 0.40 | 0.48 | 0.17 | 0.03 | 0.29 |  |
| ST40 | 20 | 3.95 | 2.24 | 0.36 | 0.48 | 0.26 | 0.12 | 0.39 |  |
| ST42 | 20 | 3.91 | 2.14 | 0.36 | 0.44 | 0.20 | 0.09 | 0.30 |  |
| ST44 | 20 | 4.67 | 2.30 | 0.39 | 0.49 | 0.21 | 0.08 | 0.34 |  |
| ST47 | 20 | 4.57 | 2.45 | 0.34 | 0.48 | 0.29 | 0.17 | 0.40 |  |
| ST50 | 20 | 4.52 | 2.39 | 0.39 | 0.46 | 0.15 | 0.04 | 0.26 |  |
| ST53 | 20 | 4.33 | 2.43 | 0.37 | 0.46 | 0.20 | 0.09 | 0.30 |  |
| ST55 | 20 | 4.10 | 2.23 | 0.36 | 0.46 | 0.23 | 0.11 | 0.35 |  |
| ST58 | 20 | 4.71 | 2.41 | 0.41 | 0.50 | 0.17 | 0.06 | 0.27 |  |
| ST59 | 20 | 4.67 | 2.39 | 0.43 | 0.49 | 0.12 | -0.01 | 0.24 |  |
| ST61 | 19 | 3.57 | 2.19 | 0.28 | 0.45 | 0.37 | 0.27 | 0.47 |  |
| ST63 | 18 | 4.14 | 2.43 | 0.38 | 0.50 | 0.25 | 0.11 | 0.37 |  |
| Mean | 19.57 | 4.27 | 2.34 | 0.36 | 0.47 | 0.23 | - | - |  |

*Population genetic structure*

**Table S3** Analysis of molecular variance (AMOVA) results for Nellie Humps demonstrating the distribution of variation across three hierarchical levels: among sampling points, among individuals within sites, and within individuals of Nellie Humps. The fixation indices that quantify genetic structure at each level are presented along with the *p* values

| **Source of variation** | **d.f.** | **Sum of squares** | **Variance components** | **Percentage of variation** | **Fixation indices** | ***p* value** |
| --- | --- | --- | --- | --- | --- | --- |
| Among sampling points | 23 | 327.10 | 0.25 | 5.36 | 0.05 | < 0.001 |
| Among individuals within sites | 406 | 2,161.07 | 0.94 | 20.22 | 0.21 | < 0.001 |
| Within individuals | 430 | 1,483.00 | 3.45 | 74.42 | 0.26 | < 0.001 |
| Total | 859 | 3,971.17 | 4.63 | - | - | - |

**Table S4** Analysis of molecular variance (AMOVA) results for SR demonstrating the distribution of variation across three hierarchical levels: among sampling points, among individuals within sites, and within individuals of Skua Ridge. The fixation indices that quantify genetic structure at each level are presented along with the *p* values

| **Source of variation** | **d.f.** | **Sum of squares** | **Variance components** | **Percentage of variation** | **Fixation indices** | ***p* value** |
| --- | --- | --- | --- | --- | --- | --- |
| Among sampling points | 20 | 248.68 | 0.17 | 3.29 | 0.03 | < 0.001 |
| Among individuals within sites | 390 | 2,329.00 | 1.12 | 22.36 | 0.23 | < 0.001 |
| Within individuals | 411 | 1,532.50 | 3.73 | 74.35 | 0.26 | < 0.001 |
| Total | 821 | 4,110.18 | 5.01 | - | - | - |

*Testing the effects of landscape heterogeneity on genetic patterns using generalised linear models (GLM)*

**Table S5** Summary of the univariate generalised linear models for collinearity between genetic kinships and various landscape explanatory variables for the Nellie Humps sampling site. The model order corresponds to their ranking from the best (smallest AIC) to worst (larger AIC) models, with the best-fit model bold highlighted. F*ij*: genetic kinship; eucl: Euclidean distance; surf: surface distance; resistance_DSM: resistance results from digital surface model analysis; *p* value: *p* value significance (‘***’ < 0.001, ‘**’ < 0.01, ‘*’ < 0.05)

| **Univariate models – Nellie Humps** | | **Predictor variable** | **Estimate** | **SE** | ***t* value** | ***p* value** | **AIC** | **ΔAIC** |
| --- | --- | --- | --- | --- | --- | --- | --- | --- |
| **1** | **F*ij* ~ resistance_DSM** | **Intercept**  **Resistance_DSM** | **0.074551**  **-3.77055** | **0.001464**  **0.072406** | **50.93**  **-52.08** | ******* | **-13,4461.9** | **0** |
| 2 | F*ij* ~ eucl | Intercept  Euclidean distance | 3.47E-02  -1.02E-04 | 7.79E-04  2.05E-06 | 44.53  -49.76 | *** | -13,4232.5 | 229.4 |
| 3 | F*ij* ~ surf | Intercept  Surface distance | 3.46E-02  -9.97E-05 | 7.78E-04  2.00E-06 | 44.51  -49.75 | *** | -13,4231.6 | 230.3 |
| 4 | F*ij* ~ topographical_barrier | Intercept  Topographical barrier | 0.007903  -0.0253914 | 0.0004554  0.0008733 | 17.35  -29.07 | *** | -13,2630.5 | 1,831.4 |
| 5 | F*ij* ~ barriers_cooling_ridge | Intercept  Barriers_cooling_ridge | 0.0075393  -0.0233667 | 0.0004582  0.0008661 | 16.45  -26.98 | *** | -13,2514.1 | 1,947.8 |
| 6 | F*ij* (Null model) | Intercept | 0.000998 | 0.00039 | 2.556 | * | -13,1791 | 2,670.9 |

**Table S6** Summary of the univariate generalised linear models for collinearity between genetic kinships and various landscape explanatory variables for Skua Ridge. The model order corresponds to their ranking from the best (smallest AIC) to worst (larger AIC) models, with the best-fit model bold highlighted. F*ij*: genetic kinship; eucl: Euclidean distance; surf: surface distance; resistance_DSM: resistance results from digital surface model analysis; *p* value: *p* value significance (‘***’ < 0.001, ‘**’ < 0.01, ‘*’ < 0.05)

| **Univariate models – Skua Ridge** | | **Predictor variable** | **Estimate** | **SE** | ***t* value** | ***p* value** | **AIC** | **ΔAIC** |
| --- | --- | --- | --- | --- | --- | --- | --- | --- |
| **1** | **F*ij* ~ resistance_DSM** | **Intercept**  **Resistance_DSM** | **3.53E-02**  **-2.52E+00** | **1.21E-03**  **8.52E-02** | **29.15**  **-29.64** | ******* | **-14,3798.6** | **0** |
| 2 | F*ij* ~ grey_lava | Intercept  Grey lava | 0.009474  -0.01704 | 0.0005027  0.000713 | 18.85  -23.91 | *** | -14,3494.7 | 303.9 |
| 3 | F*ij* ~ barriers | Intercept  Barriers | 0.010194  -0.01568 | 0.000555  0.000724 | 18.38  -21.66 | *** | -14,3392.5 | 406.1 |
| 4 | F*ij* ~ eucl | Intercept  Euclidean distance | 1.19E-02  -1.96E-05 | 7.03E-04  1.08E-06 | 16.98  -18.06 | *** | -14,3250.4 | 548.2 |
| 5 | F*ij* ~ surf | Intercept  Surface distance | 1.19E-02  -1.91E-05 | 7.02E-04  1.06E-06 | 16.94  -18.02 | *** | -14,3249.0 | 549.6 |
| 6 | F*ij* (Null model) | Intercept | 9.96E-04 | 3.58E-04 | 2.785 | ** | -14,2926.8 | 871.8 |

**Table S7** Summary of the multivariate generalised linear models for collinearity between genetic kinships and various landscape explanatory variables for the Nellie Humps sampling site. The model order corresponds to their ranking from the best (smallest AIC) to worst (larger AIC) models, with the best-fit model bold highlighted. F*ij*: genetic kinship; eucl: Euclidean distance; surf: surface distance; resistance_DSM: resistance results from digital surface model analysis; *p* value: *p* value significance (‘***’ < 0.001, ‘**’ < 0.01, ‘*’ < 0.05)

| **Multivariate models – Nellie Humps** | | **Predictor variable** | **Estimate** | **SE** | ***t* value** | ***p* value** | **AIC** | **ΔAIC** |
| --- | --- | --- | --- | --- | --- | --- | --- | --- |
| 1 | **F*ij* ~ resistance_DSM + topographical_barrier** | **Intercept**  **Resistance_DSM**  **Topographical barrier** | 8.00E-02  -3.71E+00  -2.42E-02 | 1.47E-03  0.0721279  8.61E-04 | 54.42  -51.51  -28.06 | *** | -135244.1 | **0** |
| 2 | F*ij* ~ resistance_DSM + barriers_cooling_ridge | Intercept  Resistance_dsm  Barriers_cooling_ridge | 8.05E-02  -3.75E+00  -0.02282 | 1.48E-03  0.0721345  0.0008538 | 54.56  -51.94  -26.73 | *** | -135171.5 | 72.6 |
| 3 | F*ij* ~ resistance_DSM + surf | Intercept  Resistance_DSM  Surface distance | 6.567e-02  -2.436e+00  -5.080e-05 | 1.547e-03  1.051e-01  2.903e-06 | 42.45  -23.19  -17.50 | *** | -13,4765.7 | 478.4 |
| 4 | F*ij* ~ resistance_DSM + eucl | Intercept  Resistance_DSM  Euclidean distance | 6.565e-02  -2.435e+00  -5.186e-05 | 1.548e-03  1.054e-01  2.976e-06 | 42.40  -23.11  -17.43 | *** | -13,4763.2 | 480.9 |

| **Multivariate models – Skua Ridge** | | **Predictor variable** | **Estimate** | **SE** | ***t* value** | ***p* value** | **AIC** | **ΔAIC** |
| --- | --- | --- | --- | --- | --- | --- | --- | --- |
| **1** | **F*ij* ~ resistance_DSM + grey_lava** | **Intercept**  **Resistance_DSM**  **Grey lava** | **0.0352636**  **-2.0963359**  **-0.0116881** | **0.0012078**  **0.0893305**  **0.0007461** | **29.20**  **-23.47**  **-15.66** | ******* | **-144041.6** | **0** |
| 2 | F*ij* ~ resistance_DSM + barriers | Intercept  Resistance_DSM  Barriers | 0.0349079  -2.1261880  -0.0086153 | 0.0012091  0.0925111  0.0007845 | 28.87  -22.98  -10.98 | *** | -143917.1 | 124.5 |
| 3 | F*ij* ~ resistance_DSM + river | Intercept  Resistance_DSM  River | 3.96E-02  -2.98E+00  1.16E-02 | 1.27E-03  9.51E-02  1.06E-03 | 31.11  -31.4  10.88 | *** | -14,3914.9 | 126.7 |
| 4 | F*ij* ~ resistance_DSM + surf | Intercept  Resistance_DSM  Surface distance | 3.852e-02  -3.271e+00  1.206e-05 | 1.289e-03  1.330e-01  1.648e-06 | 29.886  -24.600  7.313 | *** | -14,3850.1 | 191.5 |
| 5 | F*ij* ~ resistance_DSM + eucl | Intercept  Resistance_DSM  Euclidean distance | 3.849e-02  -3.268e+00  1.227e-05 | 1.289e-03  1.331e-01  1.688e-06 | 29.874  -24.559  7.271 | *** | -14,3849.5 | 192.1 |
| 6 | F*ij* ~ resistance_DSM + black_lava_3 | Intercept  Resistance_DSM  Black lava 3 | 0.0355129  -2.4230697  -0.0053110 | 0.0012098  0.0864475  0.0007829 | 29.354  -28.029  -6.784 | *** | -143842.6 | 199.0 |
| 7 | F*ij* ~ reistance_DSM + footpath | Intercept  Resistance_DSM  Footpath | 3.50E-02  -2.43E+00  -2.91E-03 | 1.21E-03  8.89E-02  7.74E-04 | 28.893  -27.31  -3.762 | *** | -14,3810.7 | 230.9 |
| 8 | F*ij* ~ resistance_DSM + black_lava_2 | Intercept  Resistance_DSM  Black lava 2 | 0.0352322  -2.5134126  -0.0003011 | 0.0012111  0.0894432  0.0007605 | 29.091  -28.101  -0.396 | *** | -14,3796.8 | 244.8 |

**Table S8** Summary of the multivariate generalised linear models for collinearity between genetic kinships and various landscape explanatory variables for Skua Ridge. The model order corresponds to their ranking from the best (smallest AIC) to worst (larger AIC) models, with the best-fit model bold highlighted. F*ij*: genetic kinship; eucl: Euclidean distance; surf: surface distance; resistance_DSM: resistance results from digital surface model analysis; *p* value: *p* value significance (‘***’ < 0.001, ‘**’ < 0.01, ‘*’ < 0.05)
